# The Contribution of Viral Genotype to Plasma Viral Set-Point in HIV Infection

**DOI:** 10.1371/journal.ppat.1004112

**Published:** 2014-05-01

**Authors:** Emma Hodcroft, Jarrod D. Hadfield, Esther Fearnhill, Andrew Phillips, David Dunn, Siobhan O'Shea, Deenan Pillay, Andrew J. Leigh Brown, on behalf of the UK HIV Drug Resistance Database and the UK CHIC Study

**Affiliations:** 1 Institute of Evolutionary Biology, University of Edinburgh, Ashworth Laboratories, Edinburgh, United Kingdom; 2 MRC Clinical Trials Unit Aviation House, London, United Kingdom; 3 Infection and Population Health, University College London, Royal Free Hospital, London, United Kingdom; 4 Department of Infectious Diseases, King's College London, London, United Kingdom; 5 Research Department of Infection, University College London, London, United Kingdom; University of Arizona, United States of America

## Abstract

Disease progression in HIV-infected individuals varies greatly, and while the environmental and host factors influencing this variation have been widely investigated, the viral contribution to variation in set-point viral load, a predictor of disease progression, is less clear. Previous studies, using transmission-pairs and analysis of phylogenetic signal in small numbers of individuals, have produced a wide range of viral genetic effect estimates. Here we present a novel application of a population-scale method based in quantitative genetics to estimate the viral genetic effect on set-point viral load in the UK subtype B HIV-1 epidemic, based on a very large data set. Analyzing the initial viral load and associated *pol* sequence, both taken before anti-retroviral therapy, of 8,483 patients, we estimate the proportion of variance in viral load explained by viral genetic effects to be 5.7% (CI 2.8–8.6%). We also estimated the change in viral load over time due to selection on the virus and environmental effects to be a decline of 0.05 log_10_ copies/mL/year, in contrast to recent studies which suggested a reported small increase in viral load over the last 20 years might be due to evolutionary changes in the virus. Our results suggest that in the UK epidemic, subtype B has a small but significant viral genetic effect on viral load. By allowing the analysis of large sample sizes, we expect our approach to be applicable to the estimation of the genetic contribution to traits in many organisms.

## Author Summary

HIV viral load, the amount of virus in the blood, is an important predictor of rate of CD4+ cell decline, time to AIDS and onwards transmission. Plasma viral load is influenced by many environmental and host factors, but the contribution of the viral genome is not yet clear. We have adapted a method from quantitative genetics which considers the viral phylogeny as a pedigree, permitting analysis of large cohort-derived datasets for the first time. We found the viral genome contributes significantly to the level of the set point viral load, but only determines about 6% of the variation in this property in this population. Our study also suggests that the change over time in mean plasma viral load described in some recent studies has not been due to a change in the component of viral load that is contributed by viral genotype.

## Introduction

Plasma viral load has long been considered one of the most important clinical measures in HIV-positive patients. The progression time from infection to AIDS or death varies enormously from a few years to decades, and ‘set-point’ viral load, taken early in the asymptomatic phase of the disease, is the best known early predictor of the long-term rate of disease progression [1]–[3] and is also strongly associated with transmission risk [4]–[6]. Variation in host genes, particularly HLA [7]–[12] but also the CCR5 co-receptor and its ligands, and even the gene APOBEC3G [13]–[15], has been identified as influencing progression rate, but the contribution of the viral genome is still much less clear.

Nevertheless, the hypothesis that HIV could be evolving to become more virulent has been a driver for decades of HIV research. In the mid-1980's, it became clear that some HIV isolates, deemed ‘high/fast’ lines, had a much higher replicative capacity in cell lines than others [16]–[18]. When a drop in CD4+ cell count at diagnosis was reported a few years later [19], [20], speculation began as to whether the spread of these ‘high/fast’ lines could be responsible [20]–[22]. A number of studies looking at long-term trends in HIV virulence were published, drawing mixed conclusions on whether there was evidence of HIV becoming more virulent [23]–[35]. However, a lack of standardization of when measurements were taken, what measures were used, and whether patients were on anti-retroviral therapy (ART), as well as differences in the subtypes, risk groups, and demographics of the patients involved mean that these studies are difficult to compare directly. Despite this, two meta-analyses have been performed, both concluding that a decrease in CD4+ count and an increase in viral load can be observed, implying an increase in HIV virulence that both papers suggest could be caused by viral factors [36], [37]. This would require that the viral genome exerted some influence over the set-point viral load. In the context of drug resistance it is well known that viral variation affects the replication capacity of HIV (reviewed in [38]), suggesting that such a viral genetic influence could indeed be possible.

Evolutionary theory predicts that pathogens evolve to modulate their density within hosts in order to maximize transmission rate. In the classic studies of myxomatosis [39], viral genotypes with reduced replication rates that permitted longer host survival were selected for when host density, and thus transmission probability, declined as the epidemic progressed. This, along with classic studies on the link between transmission and virulence [40], [41], raises the possibility that in the 100 years HIV is known to have infected humans [42], [43], it might have adapted to different levels of transmission probability associated with different infected populations [2], [6]. Studies of disease progression and viral load have found evidence of differences between HIV-1 subtypes [44]–[46], suggesting that major viral genetic differences among immunodeficiency viruses influence virulence.

Three studies investigated the contribution of viral genotype to set-point in studies of HIV sero-discordant couples. In these studies of 115, 56, and 47 sequence verified transmission-pairs in Zambia, the Netherlands and the USA, correlation coefficients of 0.21, 0.25, and 0.55, respectively, were estimated between set-point viral load in the index and contact cases [10], [47], [48]. Another transmission-pair study on 28 couples from Uganda reported the coefficient of determination from ANOVA analysis as *R^2^* = 27%, and *R^2^* = 37% after adjusting for confounding effects [49]. In a fourth study, based on the Swiss Cohort, Alizon et al. [50] adopted a phylogenetic approach, looking for a signal of inherited viral effect in men who have sex with men (MSM) infected with subtype B. Phylogenetic signal measures the amount that the connections in a phylogeny explain the similarity in trait values seen in different individuals [51], [52]. Using their strictest definition of set-point viral load and consequently their smallest sample size (n = 134), Alizon et al. [50] obtained a statistically significant estimate that approximately half of the variation observed in viral load could be explained by viral genetic effects. However, the estimates obtained using a more liberal definition of set-point viral load in the MSM group (n = 404) were much lower, at around 11%, and in the largest datasets where all risk-groups were included the estimates were non-significant.

Given the small numbers in all of these studies, we sought an alternative approach which would allow the inclusion of the large numbers of individuals for which both plasma viral load and viral sequence data are now available.

In quantitative genetics the proportion of the total trait variation (*V_P_*) caused by additive genetic factors (*V_A_*) is described as its narrow-sense heritability (*h^2^*). Numerous approaches have been proposed to estimate variance components and heritability from phylogenetic data, including restricted maximum-likelihood (REML) [53], maximum-likelihood (ML) [51], and generalized least squares [52]. REML methods have emerged as the preferred choice for variance component and heritability estimation due to their ability to give unbiased estimates [54]–[56]. In 1996 the program ASReml introduced an efficient implementation of REML-based variance estimation specifically designed for data from pedigreed individuals [56], [57]. By measuring the relationships between individuals on the pedigree as the probability that their alleles are identical by descent, and linking this to the observed differences in trait measures, the amount of trait variation explained by the genetic relationships can be estimated. These identical by descent relationship measures are calculated from the pedigree to form a genetic relatedness matrix, usually referred to as **A** [58].

For a phylogeny, the phylogenetic covariance of two taxa is proportional to the total length from the taxa's most recent common ancestor (MRCA) to the root under a Brownian motion model of evolution [59], [60], and the covariances between all taxa can be represented by the matrix **A**. In order to calculate variance components the inverse of **A**, **A^−1^**, is usually needed, but can be computationally resource intensive to calculate [56], [58]. Henderson [58] showed that for pedigrees this problem can be made easier by including ‘phantom parents’ for all individuals with unknown parentage so that the population could be traced back to unrelated ancestors. Hadfield and Nakagawa [56] extended this technique to phylogenies by expanding **A** to include all the internal nodes in the tree, allowing the inverse matrix to be calculated by the method of Henderson [58] and to provide a structure to the model that can be exploited by generic sparse matrix algorithms [61]. (See [62] for an alternative algorithm.)

Here, we apply this approach by using ASReml to estimate the heritability of viral load in the UK subtype B HIV epidemic, analyzing set-point viral load in almost 8,500 individuals for whom matched HIV sequences and viral load data were available.

## Results

The sequences used were made available by the UK HIV Drug Resistance Database (UK HIV RDB), which collects *pol* sequences from HIV-positive patients attending clinics across the UK before starting and during ART in order to detect resistance mutations. The UK HIV RDB was estimated to contain sequences for approximately two-thirds of the subtype B MSM patients who were treated for HIV in the UK in 2006 [63]. The first sequence available for each patient was analyzed. Fully anonymized clinical data corresponding to many of the sequences was made available by the UK Clinical HIV Cohort (UK CHIC) [64], with viral load before starting ART being available for 8,700 initial subtype B sequences, reflecting the most prevalent subtype epidemic in the UK. The data used were the most current available, with sequences and clinical data collected up to mid-2009.

After removing all cases where there was uncertainty over disease or treatment status or large sections of sequence were missing, 8,483 subtype B sequences and associated viral load measurements remained. The demographics of the dataset show that 73% (6,198 individuals) were white MSM, reflecting the historic preponderance of this subtype among MSM (Table 1). A phylogeny of these sequences was generated using RAxML [65], [66] with 38 subtype reference *pol* sequences from the Los Alamos HIV Database (www.hiv.lanl.gov) used as an outgroup.

**Table 1:** Demographics of patients whose samples were analyzed.

		Subtype B (*n* = 8,483)
Age at Set-point (years) (mean, range):		35.4 (15–83)
Log10 Set-point Viral Load (mean, SD):		4.493±0.86
**Sex**	Female:	464 *(5.5%)*
	Male:	8019 *(94.5%)*
**Risk Group**	Homo/Bisexual:	7278 *(85.8%)*
	Heterosexual:	711 *(8.4%)*
	IDU:	239 *(2.8%)*
	Other/Unknown:	255 *(3.0%)*
**Ethnicity**	White:	6990 *(82.4%)*
	Black:	597 *(7.0%)*
	Asian:	221 *(2.6%)*
	Other/Unknown:	675 *(8.0%)*

Preliminary runs in ASReml were used to determine the fixed and random effects for the model. Sex, ethnicity, country of origin, age when the set-point viral load was taken, year of HIV diagnosis, and time from HIV diagnosis to the date when set-point viral load was taken, were all included in the final model (effect estimates given in Table S1). Set-point viral load was found to increase with age, but decrease with a more recent year of diagnosis and with a longer time period between HIV diagnosis and viral load testing. HIV-positive females and non-white individuals were found to have decreased set-point viral load measures compared to males and white individuals. The random effects were estimated to have a variance of 3.11×10^−3^ and 6.55×10^−4^ for year of HIV diagnosis and country of origin, respectively.

To confirm that our method performed as expected when tested on trees with known heritabilities we performed a simulation analysis similar to that of Alizon et al. [50]. We found the estimated heritability values to correspond well with the simulated values (see Text S1).

Bootstrapped phylogenetic trees were reconstructed in duplicate on the 8,483 sequences and both trees analyzed using ASReml independently. Using the comparison of the resulting log-likelihood values from running the model with and without the tree to estimate significance, both replicates produced highly significant (p<0.0001) heritability estimates of 5.8% (CI 2.9–8.7%) and 5.6% (CI 2.6–8.5%; Table 2).

**Table 2:** Estimates of viral genetic influence on set-point viral load in HIV subtype B in the UK.

Dataset	Method	*N*	Replicate	Viral Heritability (Conf. Interval)	
Full dataset	RAxML	8,483	1	5.8%	(2.9–8.7%)
			2	5.6%	(2.6–8.5%)
Nodes with bootstraps <90% collapsed	RAxML	8,483	1	5.1%	(2.4–7.8%)
			2	6.0%	(3.1–8.8%)
BEAST 652 Sub-Sample	BEAST	652	1	5.1%	(0–11.2%)
1,726 sequences with only 1 viral load removed	RAxML	6,757	1	7.8%	(4.3–11.3%)
			2	6.6%	3.4–9.9%)

As is typical for phylogenies based on population samples of HIV *pol* sequences, there is relatively little well-supported internal structure. In order to avoid possible bias in the heritability estimates, the analysis was repeated after splits with bootstrap-support values less than 90% were collapsed (Fig. S1), which removed 78% of internal nodes. Nevertheless, the heritability estimates remained significant in each case, with estimates of 5.1% (CI 2.4–7.8%) and 6.0% (CI 3.1–8.8%). However, when the entire tree was collapsed (excepting the split to the outgroup) leaving only branch-length information, the estimate was not significant, highlighting that detecting the heritability signal relies on at least some tree structure. One hundred bootstrapped phylogenies were analyzed to further examine the effect of uncertainty in the tree. Only four of the resulting heritability estimates failed to reach significance after Bonferroni correction (though their p-values were still <0.002), resulting in a mean heritability estimate of 5.5% (CI 2.6–8.5%) (data not shown).

**Figure 1:**
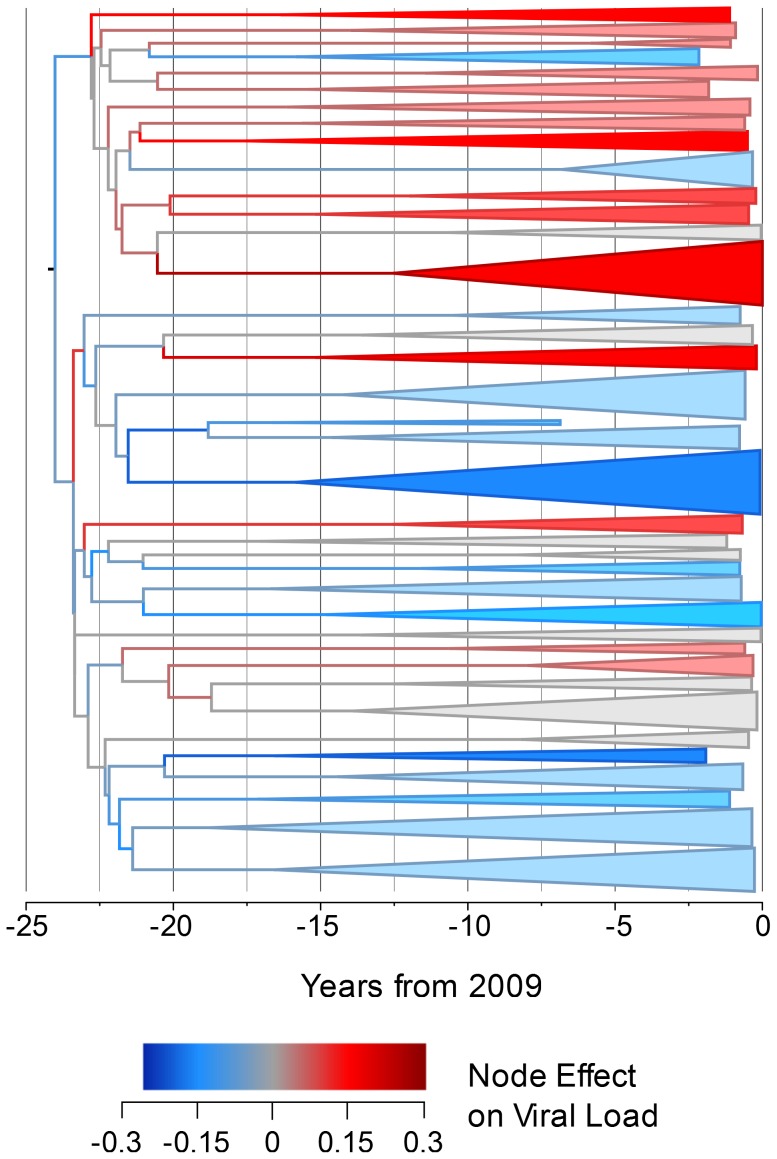
The estimated node effect plotted onto the phylogeny. The estimated phylogenetic effect of each node on log_10_ viral load plotted back onto the phylogeny from the 652-sample BEAST analysis. The axis shows the time in years from the most recent sequence, which was taken in 2009. Branches have been colored by the scale of the effect. Clusters of branches have been collapsed to improve readability, and are colored by the average tip effect within each cluster. As the number of bifurcations in the tree reduces at around 17.5 years before 2009, this used as the threshold for collapsing. Nodes that have a similar effect on viral load cluster together, as expected if some of the variation in viral load is heritable.

In order to investigate how the viral genetic effect on set-point viral load varies across the phylogeny and through time, we constructed a time-resolved phylogeny using BEAST [67]. For reasons of computational tractability, this phylogeny had to be generated on a 652-sequence sub-sample of the dataset but produced a significant heritability estimate of 5.1% (upper CI 11.2%, p<0.005). We then used ASReml to estimate the phylogenetic effect of each node on viral load and mapped these estimates onto the time-resolved phylogeny, allowing the distribution of the effects across the tree and over time to be visualized. This showed some viral lineages to be clearly associated with substantial positive genetic effects on viral load, relative to the mean, and others to be associated with equally large negative effects (Fig. 1).

**Figure 2:**
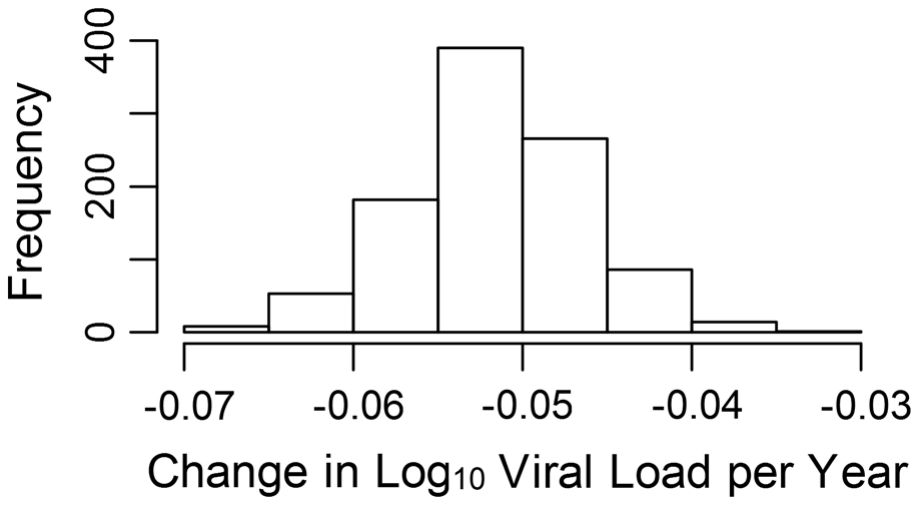
Change in viral load over time due to selection. The estimated log_10_ change in viral load per year due to selection and environmental effects (see also Fig. S2).

To investigate more formally the change in set-point viral load over time, we conducted an analysis in the R package MCMCglmm [68], [69] in order to estimate the change in viral load due to selection on the virus and environmental effects using information from the temporal variation in sample dates (see Text S2). Analysis of the change due to selection on the virus and environmental effects revealed that this would have contributed a small but significant negative change in viral load of −0.05 log_10_ copies/mL/year (Fig. 2).

## Discussion

Our analysis showed that viral genotype has a small but significant effect on set-point viral load in this population, with an estimated mean heritability of 5.7% (CI 2.8–8.6%). When the analysis was repeated after subsampling and using a different phylogenetic method, the heritability remained significant and did not differ significantly from the original estimate. As the star-like structure of HIV phylogenies can cause poor resolution of the internal nodes, resulting in low split support values, the impact of this effect was tested by collapsing weakly-supported nodes and analyzing one hundred bootstrapped phylogenies. This showed that the heritability estimates and their significance were not due to spurious or poorly-supported splits. Finally, a simulation analysis following the method of Alizon et al. [50] confirmed that our method of estimating heritability down a phylogeny performed as expected on a phylogeny where heritability is known (Text S1).

Analyzing smaller sampled datasets in BEAST allowed further investigation of the genetic effect on viral load. Plotting the estimated node effect on viral load back onto the phylogeny for the 652 sampled sequences illustrates the association of closely related sequences and similar genetic effects on viral loads in transmission chains that seem to have begun differentiating around the time subtype B arrived in the UK [70]. Finding viral lineages with both positive and negative genetic effects on viral load indicates that there is viral genetic variation that acts to both increase and decrease viral load relative to the mean.

Our estimates of the fixed effects influencing set-point viral load reflect previous reports identifying age [71], [72] and sex [73]–[75] as significant, with older individuals and males having higher set-point viral loads. We also found ethnicity to have a significant effect on set-point viral load, finding a similar estimate for the effect of Black-African ethnicity to a previous paper looking specifically at this effect [76]. Although many previous studies on the influence of ethnicity on set-point viral load suggest there is no difference between ethnic groups or that non-white minorities have higher viral loads [77]–[79], differences in socio-economic status, risk-group, and access to care make the effect of ethnicity difficult to investigate [79]. Our finding that those with a longer time from HIV diagnosis to viral load testing had a slightly lower set-point viral load could reflect that individuals with lower viral load progress more slowly and therefore may be in general slower to access care, and also indicates that we are not classifying late-stage, rising viral loads as ‘set-point,’ which would result in the opposite effect. Finally, the fact that individuals with a more recent year of diagnosis also have a slightly lower set-point viral load could suggest that the proportion of individuals being diagnosed in late-stage infection is decreasing with time [80].

Previous studies investigating the heritability of viral load in HIV have estimated the genetic effect at between 11 to 60%, higher than the estimate of 5.7% obtained here. Three of the five studies were done on cohorts infected with subtypes other than B; one on subtype C [10] and two on mixed subtype populations [47], [49], making comparisons difficult. Because virulence differs between subtypes [44]–[46], heritability estimates could be affected in studies where the cohort is infected with multiple subtypes, even when subtype is included as a variable in the model. Similarly, both the environmental and genetic variance that determines heritability can vary between populations, and may be particularly divergent between studies focusing on different demographic or risk groups (see [81] for further discussion). Considering this, some disparity in heritability estimates may not be unexpected.

Four of the previous studies used transmission pairs (n = 28 to n = 115) to estimate the heritability of viral load, and this could also influence the estimates obtained. As pointed out in one of these studies [10], the sero-discordant couples where transmission does occur may not accurately reflect the epidemic as a whole. As viral load is proportional to the probability of transmission, partners who transmit HIV and thus get included in the analysis had higher viral loads than average for the study [10]. Cohabiting or long-term sexual partners may also share confounded environmental factors such as diet and exposure to other pathogens, which could affect health and thus viral load, and may even share HLA alleles, which increases HIV transmission risk and the between-partner correlation in viral load [82], [83].

The only previous study that utilized a phylogeny-based approach also reported a heritability estimate considerably higher than the one obtained here. Alizon et al. [50] obtained a significant heritability estimate of around 50% when they used the most stringent criteria to define which samples would be taken as set-point viral load. Heritability estimates apply only to the population studied, so their estimate may be specific to this small (n = 134) population of MSM individuals with exceptionally stable viral load measures. Interestingly, when they relaxed their definition of set-point viral load, tripling the sample size, the heritability estimates shrank to around 11%. More generally, heritability estimates between studies where the sequences have different times to their respective MRCA are not readily compared. Studies with a more distant MRCA are likely to have higher heritability estimates as we expect the variance of the phenotype at the tips to increase with increasing time to the MRCA.

Given that viral genotype is influencing viral load, the question arises as to the source of this effect in the viral genome. The analysis has been performed on the *pol* gene, where both drug resistant and naturally occurring variation is known to affect replicative capacity [84]. It is also possible that this between-lineage variation (Fig. 1) could be a distal effect that may map to one or more other genes, such as *env* [85], that we are detecting through its linkage with variants in the *pol* gene. With increasing availability of full-genome datasets it may be possible to address that question directly in future.

The analysis performed here avoided issues associated with using multiple subtypes, transmission pairs, or restricted samples by including as many cases as possible. The aim was to minimize bias, but this clearly would be expected to introduce a substantial amount of noise and depends on the availability of large datasets. In fact twenty-fold more individuals were included than the largest previous dataset with a significant heritability estimate [50]. Nevertheless, this approach could allow some viral loads to be classified as set-point when they were actually taken during the acute stage, prior to the onset of AIDS, while on ART, or during a transient rise in viral load. The data cleaning methods utilized were able to exclude several cases that may have fallen into these categories, but this was difficult when there was only one pre-ART measure, as applied to approximately 20% of the dataset (1,726 cases). If many of the viral loads classified as set-point are not actually set-point measurements, this could affect the estimate of heritability obtained. However, when the dataset was re-run after removing these 1,726 cases, the heritability estimates remained significant with a mean value of 7.2% (CI 3.9–10.6%), showing that any errors made in classifying sequences with just one pre-ART viral load do not significantly affect the estimate.

We found no evidence that subtype B HIV is becoming more virulent in the UK. Indeed, the relatively small heritability of around 6% implies that host, environmental, and demographic effects play a much larger role in determining viral load than the virus genotype in this population and suggests that any change in viral load due to the viral genotype would be relatively small. The implications of a heritable viral load have been extensively explored, especially in the context of HIV adapting towards an ‘optimal’ viral load for transmission due to selection [2], [81]. Our findings, however, imply that selection on the viral genetic component of viral load would have very limited influence on viral evolution.

The MCMCglmm analysis estimated a small but significant decrease over time of −0.05 log_10_ copies/mL/year in the mean value of the component of viral load determined by viral genotype (see Text S2). At this time the change due to selection on the virus cannot be disentangled from change to due environmental effects we have not controlled for, such as the background level of ART in the population, so we cannot assume all (or even any) of this change is due to selection on the viral genome. It should also be noted that though the viral genetic influence on viral load seems to be causing a decrease in viral load, this does not necessarily mean that overall viral load would be expected to decrease. As we estimate the viral genetic contribution to the variance in viral load to be only about 6%, changes in any of the many host and environmental factors influencing viral load could cause viral load to remain constant or even increase.

Previous cohort-based studies of viral load data have indeed estimated an increase in the phenotypic value. In an analysis based on 1,584 individuals with viral load data from the 22 CASCADE cohorts, Dorrucci et al. [36] estimated an increase in set-point of 0.044 log_10_ copies/mL/year, leading to an increase in set-point viral load of more than a log over 30 years. Herbeck et al. [37] performed a meta-analysis based on eight previous studies investigating change in viral load, which generated a more modest estimate of 0.013 log_10_ copies/mL/year and an overall increase of 0.39 log_10_ copies/mL in 30 years. These changes have led to suggestions that the virus may have evolved to become more virulent [36], [37], but this was not directly analyzed and is clearly not the case in our study. However, a much larger fraction of the phenotypic value of viral load in our model is determined by the fixed effects including sex, age and time from diagnosis to first viral load which have certainly not remained constant over the course of the epidemic, so the two observations by no means necessarily conflict. The studies included by Herbeck et al. range from −0.013 to 0.056 log_10_ copies/mL/year in their estimates, with the largest study reporting a significant decline of −0.013 log_10_ copies/mL/year. This suggests that changes in viral load are difficult to quantify and may be quite population specific, with different environmental effects and selection pressures working in each.

Our findings indicate that the genotype of HIV subtype B in the UK has a small but significant effect on viral load, and suggest that the virulence of HIV has not increased. The use of this novel method in other situations where sequence data are available could allow estimation of heritability where it has not previously been possible.

## Methods

8,700 initial subtype B sequences from the UK HIV RDB had viral load measures before starting ART available from UK CHIC. Sequences were aligned using the Stanford HIVdb Program [86], with manual checks for high levels of ambiguity and poor quality. To maintain both the representativeness of the HIV epidemic in the UK and as large a sample size as possible to improve power, a liberal definition of set-point viral load was chosen. If multiple pre-ART viral load measures were available for a patient, the first viral load was generally taken as the ‘set-point’ viral load. To exclude viral loads taken in AIDS, while the patient was on unreported ART, or while the patient was still in the acute phase of the disease, exclusion rules were applied. Unusually low or high viral load measures (<400 copies/mL or >1×10^6^ copies/mL) were inspected for evidence of unreported ART use, acute infection, or onset of AIDS, and excluded if any of these were suggested. A full description of the rules used to discard records and the number of records removed can be found in Table S2. Set-point viral load values were log_10_ transformed before the analysis to make the distribution approximately normal. 80% of the patients included in our dataset had the viral load used as set-point taken within three years of HIV diagnosis. More information about the dates of HIV diagnosis and set-point viral load tests is available in Text S3.

Patients with accepted viral loads and at least 840 nucleotides of *pol* sequence (PR and partial RT coding regions) were analyzed. A total of 119 identical sequences from different patients were also removed, as they are likely to include multiple sequences from the same individual submitted under different identifiers. This left 8,483 subtype B sequences with matched viral loads. Sequences were stripped of codons in positions associated with drug-resistance mutations [87], [88] before phylogenetic analysis. The analysis was repeated on sequences not stripped of resistance-associated codons, but no significant differences in heritability estimates were observed. To provide an unbiased root for the tree, 38 subtype reference *pol* sequences (subtypes A-K) from the Los Alamos HIV Database (www.hiv.lanl.gov) were used as an outgroup.

The large size of the dataset limited the methods available to create the phylogenies. RAxML [65], [66] is an ML-based phylogenetic program designed to handle large alignments and produce accurate phylogenies by conducting a thorough topology search [89] and also performing bootstraps. We implemented RAxML on the Edinburgh Compute and Data Facility computer cluster. 100 bootstraps for each phylogeny were generated using the parallelized version of RAxML on 16 processors with a run time of 30 hrs. A comprehensive ML tree search was performed using the threaded version of RAxML on 12 processors for an average of 100 hrs, and the bootstrap-support values were then written onto the ML tree.

### Pipeline

A new piece of software, TreeCollapseCL 4 (available at http://hiv.bio.ed.ac.uk), was developed to aid in preparing the phylogenies for further analysis and investigation of the data. Using TreeCollapseCL 4, each phylogeny was rooted and the average length of the tree was calculated from the tips to the MRCA of the UK sequences in the dataset (the second node from the root). Branch length to the root was not calculated because the distance from the root to the MRCA of the UK HIV RDB sequences can be severely affected by the choice of outgroup used.

The sampled viral sequences and all of the internal nodes of the phylogeny were incorporated into a genetic relatedness matrix from which the inverse was calculated using the R [68] package MCMCglmm [69].

The phylogenetic covariance of two individuals on a phylogeny was assumed to be proportional to the distance between their MRCA and the root [59]. Thus the covariance of an individual with itself is its distance from the root in units of substitutions per site per year. In phylogenetic comparative methods that use ultrametric trees, the distance between the tips and the root is often rescaled to one unit. Although the units are arbitrary, the variance explained by the phylogeny is directly interpretable as the variance explained in the sample of individuals used in the analysis. However, when trees are not ultrametric, as in this case, the root-to-tip distances vary. In this instance we standardized by the average distance from root to tip of 0.14 substitutions per site per year calculated earlier by TreeCollapseCL 4.

Preliminary runs were carried out on the dataset in ASReml in order to identify the fixed effects to include in the final model. Age at the sample date taken for set-point viral load, sex, ethnicity, time from HIV diagnosis to the set-point viral load sample date, and year of HIV diagnosis (as a continuous effect) were included in the preliminary models. All of the terms were found to be highly significant (p<0.001) and therefore all were included in the final model. Country of origin and year of HIV diagnosis (as a categorical effect) were also included as random effects along with the phylogeny. Year of HIV diagnosis was included as a continuous fixed effect to model the linear change in set-point viral load and as a categorical random effect to account for any random deviations around this trend from year to year. As country of origin has many discrete levels, it was included as a random effect in order to estimate the variance of their effects.

The significance of the effect of the phylogeny in explaining the variance was assessed by first running the model without the phylogeny as a ‘null’ model and then including the phylogeny. A log-likelihood ratio test with one degree of freedom was then used to test whether the model with the phylogeny was significantly better at explaining the variation in viral load than the null model.

As ASReml assumes all pedigree information provided is correct, analyses were repeated using TreeCollapseCL 4 to collapse splits with bootstrap-support values less than 90% down to polytomies. To further evaluate how uncertainty in the tree could affect our heritability estimates, one hundred bootstrapped trees were generated in RAxML and analyzed. Because of the close phylogenetic relationship between the subtype B and D in the *pol* region bootstrapped subtype D sequences can sometimes cluster within the B clade, making it necessary to remove the subtype D Los Alamos sequences from the phylogeny in order to root all 100 trees by the same outgroup.

Each analysis was performed in duplicate, with the sequences being run through RAxML and the analysis pipeline twice. The significance threshold used was adjusted using a Bonferroni correction for the number of replicates.

In order to investigate whether set-point viral load has changed over time, we estimated the amount of change in viral load due to selection. This can be estimated using Markov chain Monte Carlo methods to calculate the total contribution of between-lineage and within-host selection, though we cannot distinguish all change due to within-host selection from environmental factors (see Text S2 for more detail).

In addition, to further investigate the phylogenetic effects on viral load, a time-scaled phylogeny was produced using BEAST, a Bayesian phylogenetic program [67]. Because the complexity of the analysis performed by BEAST limits the number of samples which can be run in a reasonable time-frame, a sub-sample of the main dataset was used.

After collapsing nodes with bootstrap support values less than 90%, 965 sequences remained in un-collapsed clusters of fifteen or more sequences. A random subsample of 652 of these sequences was taken for analysis in BEAST. BEAST was run with a relaxed log-normal clock and a constant population size for 100,000,000 steps, sampling every 10,000 steps. All runs were performed in duplicate, and after 10% burn-in was removed the resulting files were combined using an in-house script. A summary tree was then generated using the BEAST program TreeAnnotator, and run in ASReml to obtain heritability estimates.

Finally, in order to validate the heritability estimates produced by our pipeline, we followed the method of Alizon et al. [50] (see Text S1) to perform a simulation analysis where viral loads were simulated down trees under known heritabilities.

### Accession numbers

As submission of the entire UK HIV Drug Resistance Database online would risk breaching patient confidentiality by allowing transmission networks to be identified, following Kouyos et al. (J Infect Dis, 2010) and Leigh Brown et al. (J Infect Dis, 2011) a random sample of 10% of the database has been submitted to GenBank under accession numbers JN100661-JN101948.

### Ethics statement

This work was performed on data generated in the course of routine clinical care which was anonymized and delinked before analysis. Ethical approval for this work was given by the London Multicentre Research Ethics Committee (MREC/01/2/10; 5 April 2001).

### Members of the UK HIV Drug Resistance Database Steering Committee

Celia Aitken (Gartnavel General Hospital, Glasgow); David Asboe, Anton Pozniak (Chelsea & Westminster Hospital, London); Patricia Cane (Public Health England, Porton Down); Hannah Castro, David Dunn*, Esther Fearnhill, Kholoud Porter (MRC Clinical Trials Unit at UCL, London); David Chadwick (South Tees Hospitals NHS Trust, Middlesbrough); Duncan Churchill (Brighton and Sussex University Hospitals NHS Trust); Duncan Clark (St Bartholomew's and The London NHS Trust); Simon Collins (HIV i-Base, London); Valerie Delpech (Centre for Infections, Public Health England); Samuel Douthwaite (Guy's and St. Thomas' NHS Foundation Trust, London); Anna Maria Geretti (Institute of Infection and Global Health, University of Liverpool); Antony Hale (Leeds Teaching Hospitals NHS Trust); Stéphane Hué (University College London); Steve Kaye (Imperial College, London); Paul Kellam (Wellcome Trust Sanger Institute & University College London Medical School); Linda Lazarus (Expert Advisory Group on AIDS Secretariat, Public Health England); Andrew Leigh-Brown (University of Edinburgh); Tamyo Mbisa (Virus Reference Department, Public Health England); Nicola Mackie (Imperial NHS Trust, London); Chloe Orkin (St. Bartholomew's Hospital, London); Eleni Nastouli, Deenan Pillay*, Andrew Phillips, Caroline Sabin (University College London Medical School. London); Erasmus Smit (Public Health England, Birmingham Heartlands Hospital); Kate Templeton (Royal Infirmary of Edinburgh); Peter Tilston (Manchester Royal Infirmary); Daniel Webster (Royal Free NHS Trust, London); Ian Williams (Mortimer Market Centre, London); Hongyi Zhang (Addenbrooke's Hospital, Cambridge); Mark Zuckerman (King's College Hospital, London).

*Co-PI

### Members of the UK CHIC Steering Committee

Jonathan Ainsworth, North Middlesex University Hospital NHS Trust, London; Sris Allan, Coventry & Warwickshire NHS Trust; Jane Anderson, Homerton University Hospital NHS Trust, London; Abdel Babiker, MRC Clinical Trials Unit, London; David Chadwick, South Tees Hospitals NHS Foundation Trust, Middlesbrough; Valerie Delpech, Health Protection Agency Centre for Infections (HPA CfI), London; David Dunn, MRC Clinical Trials Unit, London; Martin Fisher, Brighton and Sussex University Hospitals NHS Trust, Brighton; Brian Gazzard (Chair), Chelsea & Westminster Hospital NHS Foundation Trust, London; Richard Gilson, Mortimer Market Centre, University College London Medical School; Mark Gompels, North Bristol NHS Trust, Bristol; Phillip Hay, St George's Healthcare NHS Trust, London; Teresa Hill, University College London Medical School; Margaret Johnson, Royal Free Hampstead NHS Trust, London; Stephen Kegg, South London Healthcare NHS Trust, London; Clifford Leen, The Lothian University Hospitals NHS Trust, Edinburgh; Fabiola Martin, York Teaching Hospital NHS Foundation Trust; Mark Nelson, Chelsea and Westminster Hospital NHS Foundation Trust, London; Chloe Orkin, Barts and The London NHS Trust, London; Adrian Palfreeman, University Hospitals of Leicester NHS Trust; Andrew Phillips, University College London Medical School; Deenan Pillay, University College London; Jillian Pritchard, Ashford & St. Peter's Hospitals NHS Foundation Trust; Frank Post, King's College Hospital NHS Foundation Trust, London; Caroline Sabin, University College London Medical School; Memory Sachikonye, UK Community Advisory; Board (UK-CAB); Achim Schwenk, North Middlesex University Hospital NHS Trust, London; Anjum Tariq, The Royal Wolverhampton NHS Trust; John Walsh, Imperial College Healthcare NHS Trust, London.

## Supporting Information

Figure S1 The effect of collapsing poorly-supported nodes. A sub-section of the full RAxML tree is shown before (A) and after (B) collapsing nodes with bootstrap support less than 90% down to polytomies. Branch length from root to tip nodes is preserved after collapsing.
(TIF)

Figure S2 The log_10_ change in viral load per year due to selection. The estimated log_10_ change in viral load per year due to between-lineage selection (shaded) and within-host selection and environmental effects (unshaded). Though the change due to between-lineage selection was not significantly different from what could be expected through drift, the change due to within-host selection and environmental effects was significant.
(TIF)

Table S1 Mean fixed effect estimates.
(PDF)

Table S2 Details of sequences removed during data cleaning.
(PDF)

Text S1 Supplementary methods detailing the simulation performed to verify the heritability estimate obtained.
(PDF)

Text S2 Within-host and between-lineage selection analysis and simulations.
(PDF)

Text S3 Additional information about time of diagnosis and viral load test date.
(PDF)
