# Supplementary figures and images for: The Contribution of Viral Genotype to Plasma Viral Set-Point in HIV Infection
Source: PLoS Pathog. 2014 May 1;10(5):e1004112. doi: 10.1371/journal.ppat.1004112 (PMC4006911; doi:10.1371/journal.ppat.1004112)

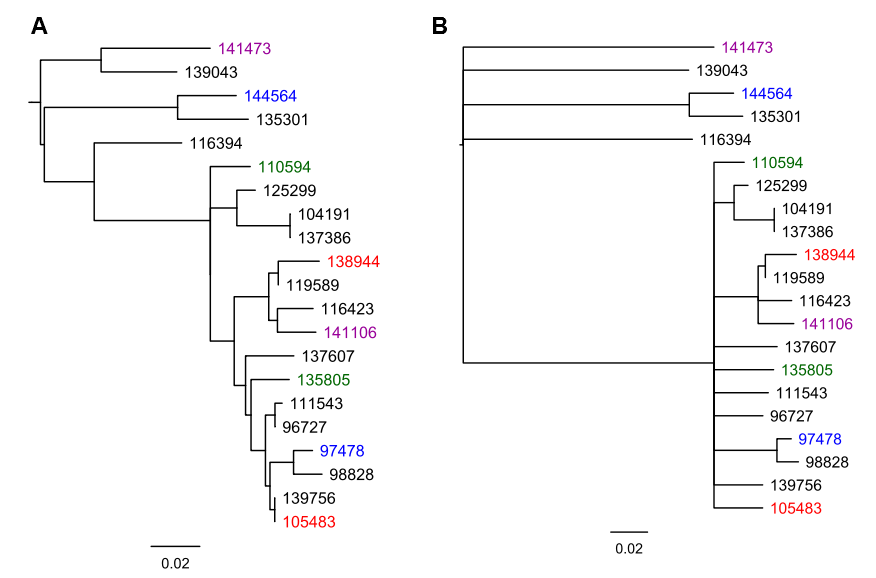

Supplement: Figure S1 — The effect of collapsing poorly-supported nodes. A sub-section of the full RAxML tree is shown before (A) and after (B) collapsing nodes with bootstrap support less than 90% down to polytomies. Branch length from root to tip nodes is preserved after collapsing. (TIF) [file ppat.1004112.s001.tif]
