# Supplementary material for: The Contribution of Viral Genotype to Plasma Viral Set-Point in HIV Infection
Source: PLoS Pathog. 2014 May 1;10(5):e1004112. doi: 10.1371/journal.ppat.1004112 (PMC4006911; doi:10.1371/journal.ppat.1004112)
Supplement: Table S1 — Mean fixed effect estimates. (PDF) [file ppat.1004112.s003.pdf]

Supplementary Information Table1 – Mean Fixed Effect Estimates of spVL Influence

Effect	Units	Estimate	Standard Error
Intercept		4.508	0.117
Age at viral load test*	per year	8.48E-03	1.13E-03
Year of HIV Diagnosis*	per year	-4.00E-03	3.46E-03
Time from HIV Diagnosis to viral load test*	per day	-6.18E-05	1.49E-05
Sex	Male	0	0
	Female	-0.200	0.044
Ethnicity	White	0	0
	Asian/Oriental	-0.038	0.076
	Black-other	-0.127	0.073
	Indian/Pakistani/Bangladeshi	-0.136	0.093
	Other/Mixed	-0.142	0.039
	Black-Caribbean	-0.165	0.053
	Not known	-0.171	0.079
	Black-African	-0.260	0.075
	Other	-0.267	0.161

*Values were means-adjusted before analysis
