# Supplementary material for: The Contribution of Viral Genotype to Plasma Viral Set-Point in HIV Infection
Source: PLoS Pathog. 2014 May 1;10(5):e1004112. doi: 10.1371/journal.ppat.1004112 (PMC4006911; doi:10.1371/journal.ppat.1004112)
Supplement: Table S2 — Details of sequences removed during data cleaning. (PDF) [file ppat.1004112.s004.pdf]

Supplementary Information Table 2 – Details of Sequences Removed During Data Cleaning

| | | |
|---|---|---|
| Number of subtype B sequences with at least 1 viral load measure before ART | | 8,700 |
| 1 VL measure, which is >= 1,000,000 where a CD4 is available within 1 month and is over 500 (could be in acute stage) | -12 | |
| 1 VL measure, which is 500,000 or 750,000 (measurement limit, treated as over 1,000,000) where a CD4 is available within 1 month and is over 500 (could be in acute stage) | -21 | |
| 1 VL measure, which is <=50 where a CD4 is available within 1 month and is over 200 (could be on ART) | -26 | |
| 1 VL measure, which is 400 (measurement limit, treated as being 50) where a CD4 is available within 1 month and is over 200 (could be on ART) | -3 | |
| VL measures over 1,000,000 (or 500,000 or 750,000, treated as 1,000,000) that fall below 1,000 in 93 days (could have started ART) | -8 | |
| 1 VL measure of 1, rejected as potential database error | -1 | |
| Missing 200bp in RT | -7 | |
| Identical sequences removed | -119 | |
| Missing RT or protease | -20 | |
| Final number of records | | 8,483 |
