# Supplementary material for: The Contribution of Viral Genotype to Plasma Viral Set-Point in HIV Infection
Source: PLoS Pathog. 2014 May 1;10(5):e1004112. doi: 10.1371/journal.ppat.1004112 (PMC4006911; doi:10.1371/journal.ppat.1004112)
Supplement: Text S1 — Supplementary methods detailing the simulation performed to verify the heritability estimate obtained. (PDF) [file ppat.1004112.s005.pdf]

Text S1 – Supplementary text to *The Contribution of Viral Genotype to Plama Viral Set-Point in HIV Infection*

Simulations to Assess the Method for Estimating Heritability Down a Phylogeny

In order to assess the performance of our heritability estimator under well-controlled conditions, simulations were performed using a specified heritability to generate viral load measures down a phylogeny, which was then run through our analysis pipeline.

The simulations were based on those performed by Alizon et al. (2010) [1]. In their paper, an SIR model was used to generate twenty phylogenies over thirteen generations with a probability of transmission of 0.75 (modelled by a branching in the tree), and a probability of death of 0.25. When transmission occurred, one 'child' branch retained the viral load of the 'parent,' while the viral load of the other 'child' branch was determined by the relationship:

$$x_{a+1} = \zeta x_a + (1 - \zeta)y$$

where x_{a+1} is the viral load of the new 'child', x_a is the viral load of the 'parent,' y is a random value drawn from the empirical distribution of viral loads in the population, and ζ is the heritability of viral load. (In passing, it is worth pointing out that under a Brownian motion model (assumed in our main analysis) both children would assume new viral load values according to this equation.)

We utilized the same equation in our simulations, but used the square root of the heritability and one minus the heritability in order to prevent the loss of variance that occurs at each transmission if heritability is used. Thus, equation used was:

$$x_{a+1} = \sqrt{\zeta} x_a + \sqrt{(1 - \zeta)} y$$

The phylogenies simulated for Alizon et al. (2010) [1], ranging in size from 128-700 tips, were kindly provided by Dr. Samuel Alizon. Using our modified equation, viral load was

simulated down these phylogenies at heritability values of 30%, 50%, 70%, and 90%, drawing random values (*y*) from the empirical distribution of our own B-subtype dataset. As there were twenty trees simulated by Alizon et al. for each heritability value, we simulated viral load values down each tree independently. Because the trees used by Alizon et al. were created by simulating the probability of death and transmission, this was indirectly incorporated into our analysis, though we did not explicitly model it.

The resulting phylogenies were then passed through the same pipeline as used in our main analysis; however, it was necessary to account for some of the different assumptions made in the simulation and in our main analysis.

In our main analysis, the branch lengths are of utmost importance, as they define the degree of relatedness between sequences. Branch lengths represent not only time over which the virus can change by mutation, but subsume transmission events which may exert selection on the virus. However, in the simulation branch length contributes no information as all transmissions are known, and viral load can only change at transmission events. The simulated phylogenies are more like conventional pedigrees, where the number of branching events conveys relatedness. Thus, before analysis all branch lengths on the simulated phylogenies were set to one, removing the requirement to rescale the heritability estimates by average root-to-tip distance.

After analysing each of the simulated phylogenies using our pipeline, the resulting heritability estimates were plotted (Fig S3). We found the estimated heritability values to correspond well with the simulated values, especially given the much smaller sample size of the simulated phylogenies than our actual B-subtype dataset. The simulation confirms that our method for estimating the heritability of viral load produces expected results.

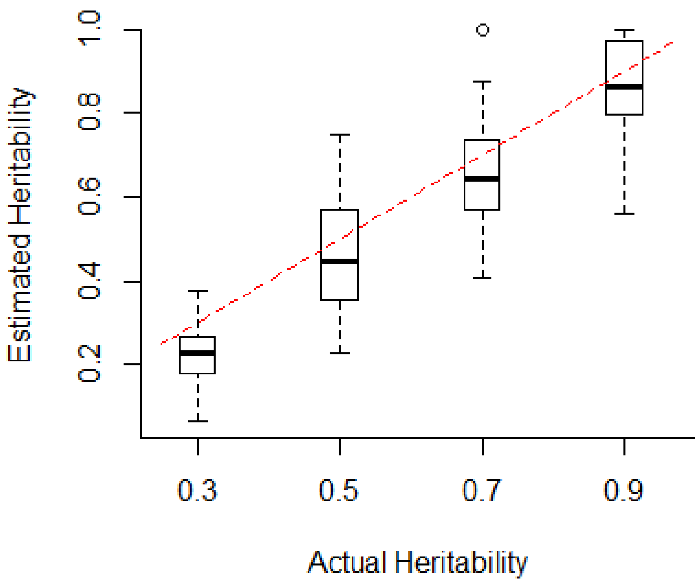

Figure S3. Heritability estimates on simulated trees with known heritability. Viral loads were simulated down 20 trees independently at each heritability value. The box-plot shows the median heritability, quartiles, and outliers for each heritability simulated. The red dashed line shows *x=y*.

References:

1. Alizon S, von Wyl V, Stadler T, Kouyos RD, Yerly S, et al. (2010) Phylogenetic Approach Reveals That Virus Genotype Largely Determines HIV Set-Point Viral Load. PLoS Pathog 6: e1001123. doi:10.1371/journal.ppat.1001123.
