# Supplementary material for: The Contribution of Viral Genotype to Plasma Viral Set-Point in HIV Infection
Source: PLoS Pathog. 2014 May 1;10(5):e1004112. doi: 10.1371/journal.ppat.1004112 (PMC4006911; doi:10.1371/journal.ppat.1004112)
Supplement: Text S2 — Within-host and between-lineage selection analysis and simulations. (PDF) [file ppat.1004112.s006.pdf]

Text S2 – Supplementary Text to *The Contribution of Viral Genotype to Plasma Viral Set-Point in HIV Infection*

Within-Host and Between-Lineage Selection Analysis and Simulations

In order to look at the change in set-point viral load due to selection, we examined the total effect of within-host and between-lineage selection. Within-host selection occurs when variation in set-point viral load determines the relative frequency of the genotype within a host. We can estimate the within-host change in longitudinal data by fitting sequence sample date as a covariate in the model, which was done using the MCMCglmm package. However, any directional change due to environmental factors not controlled for in our model that influence viral load, such as the background level of antiretroviral treatment in the population, could give rise to identical patterns, and currently we are unable to distinguish between the two.

Between-lineage selection happens when variation in set-point viral load determines the probability of transmission (speciation) and host death (extinction). In this context it is known that evolutionary change can be estimated by taking the difference in the means of predicted breeding values (the equivalent of phylogenetic effects) over time [1]. Markov chain Monte Carlo methods can be used to average over the uncertainty in the heritability estimates and the predicted breeding values in order to derive the posterior distribution of evolutionary change [2]. Posterior predictive tests can then determine whether evolutionary change has occurred and whether the change is greater than would be expected by chance (drift) [3]. Here we implemented an equivalent model on the phylogeny.

It is known that selection estimates rely on any missing data being missing at random and not dependent on the value of the data, such as when the trait value determines survival probability and thus sampling [4,5]. Because the data will not be missing at random if speciation and/or extinction is dependent on viral load, which is the case in HIV, then the

method may be expected to give biased estimates of evolutionary change. In addition, phenotyping of a pedigree is often comprehensive in comparison to the phenotyping of species in a phylogeny, where all ancestral taxa usually have missing data. Although more appropriate methods exist for this type of problem [6], the size and complexity of our data prohibit their use. To gauge the magnitude of the problem we simulated data under a model of speciation and extinction with trait-dependent rates using the make.quasse function in the Diversitree R package [7].

We simulated 500 100-tip trees with the probability of speciation or extinction either being a constant or depending on the trait through a linear model on the logit scale. The two parameters of the linear model were an intercept and a slope on trait value. In the first set of simulations extinction probability was set to zero, and speciation modeled with an intercept of zero and slope of 0.75. In the second set of simulations speciation probability was set to one, and extinction modeled with an intercept of -2 and slope of 5. The rate of drift (proportional to the phylogenetic variance) was set to 0.1, and independent random normal deviates with a variance of 0.05 added to each character. The models of speciation and extinction depend on the trait value before adding the random normal deviates (i.e. they depend on the phylogenetic effects only). The magnitudes of evolutionary change in the two sets of simulations were considerably larger than the rate of drift and roughly equal to each other (although opposing in sign).

The method was capable of detecting evolutionary change under a trait-mediated speciation model but the magnitude of the change was underestimated, with an average change across simulations of 0.359 but an average estimate of 0.295 (i.e. 82% the true value). When between-lineage evolutionary change was mediated by differential extinction the average change across simulations was -0.352 but the average estimate was -0.053 (i.e. 15% the true value). The results, together with those of analyses that included the missing phenotypes of taxa ancestral to the extant taxa (i.e. the missing data of the speciation only model) are presented in Table S3 below.

Our simulations suggest that although the power to detect evolutionary change via differential speciation was relatively good and the downward bias in the estimate of the magnitude of evolutionary change not too severe, any evolutionary change caused by differential extinction is hard to detect and its magnitude considerably underestimated. However, in HIV differential extinction and differential transmission are not easy to distinguish as viral load has a positive effect on transmission probability and a negative effect on infection duration and therefore potential for transmission [8].

Though these simulations suggest that our estimate of change due to between-lineage selection is probably an underestimate, even if the true value is double our prediction (0.002 $\log_{10}$ copies/mL/year) its magnitude is very small compared with the change due to within-host selection and environmental effects (-0.05 $\log_{10}$ copies/mL/year) (see Fig. S2). Therefore, the overall change due to selection on the virus will be largely due to this within-host component.

Table S3 - Means and standard errors of observed and estimated evolutionary change for 500 data-sets simulated under models of trait-mediated speciation and extinction. Estimate I is the estimate made when only the phenotypic data of extant taxa are observed, and estimate II is the estimate made when the phenotypic data of all ancestral nodes of extant taxa are also observed.

	Speciation	Extinction
Actual Change	0.359±0.016	-0.352±0.011
Average Estimate I	0.295±0.011	-0.053±0.006
Average Estimate II	0.377±0.014	-0.104±0.009

Supplementary Text 1 References

1. Walsh B, Lynch M (2012). Evolution and Selection of Quantitative Traits: I. Foundations. Sunderland, MA: Sinauer, Vol. I. pp. 179–208.

2. Sorensen DA, Wang CS, Jensen J, Gianola D (1994) Bayesian analysis of genetic change due to selection using Gibbs sampling. Genet Sel Evol 26: 1–28. doi:10.1186/1297-9686-26-4-333.

3. Hadfield JD, Wilson AJ, Garant D, Sheldon BC, Kruuk LEB (2010) The Misuse of BLUP in Ecology and Evolution. The American Naturalist 175: 116–125. doi:10.1086/648604.

4. Rubin DB (1976) Inference and missing data. Biometrika 63: 581–592. doi:10.1093/biomet/63.3.581.

5. Im S, Fernando R, Gianola D (1989) Likelihood inferences in animal breeding under selection: a missing-data theory view point. Genet Sel Evol 21: 399–414. doi:10.1186/1297-9686-21-4-399.

6. FitzJohn RG (2010) Quantitative Traits and Diversification. Syst Biol 59: 619–633. doi:10.1093/sysbio/syq053.

7. FitzJohn RG (2012) Diversitree: comparative phylogenetic analyses of diversification in R. Methods in Ecology and Evolution 3: 1084–1092. doi:10.1111/j.2041-210X.2012.00234.x.

8. Fraser C, Hollingsworth TD, Chapman R, de Wolf F, Hanage WP (2007) Variation in HIV-1 set-point viral load: Epidemiological analysis and an evolutionary hypothesis. Proceedings of the National Academy of Sciences 104: 17441–17446. doi:10.1073/pnas.0708559104.
