# Supplementary material for: The Contribution of Viral Genotype to Plasma Viral Set-Point in HIV Infection
Source: PLoS Pathog. 2014 May 1;10(5):e1004112. doi: 10.1371/journal.ppat.1004112 (PMC4006911; doi:10.1371/journal.ppat.1004112)
Supplement: Text S3 — Additional information about time of diagnosis and viral load test date. (PDF) [file ppat.1004112.s007.pdf]

Text S3 - Supplementary Text to *The Contribution of Viral Genotype to Plasma Viral Set-Point in HIV Infection*

Additional Information about Time of Diagnosis and Viral Load Test Date

Our dataset included patients diagnosed as HIV positive from 1980 through 2009 (Table S4). Viral load testing was only widely practiced from 1995, so most records are after this date (Fig. S4). To calculate the time between HIV diagnosis and the date of the viral load test taken as set-point, the diagnosis date is subtracted from the viral load test date (Table S5, Fig. S5). 80% of patients in the dataset had the set-point viral load test taken within 3 years of HIV diagnosis, suggesting that most viral loads taken are indeed during the chronic phase.

**Table S4** – Median, quartiles, and range of HIV diagnosis date and set-point viral load test date.

| | **1st Quartile** | **Median** | **3rd Quartile** | **Range** |
|---|---|---|---|---|
| HIV Diagnosis | 5-Oct-1998 | 17-Jun-2003 | 27-Apr-2006 | 1-Jan-1980 to 6-May-2009 |
| Date of Set-point Viral Load Test | 7-Oct-1999 | 22-Dec-2003 | 14-Aug-2006 | 29-Jul-1986 to 13-May-2009 |

**Table S5** – Median, quartiles, and range of days between HIV diagnosis and set-point viral load test date.

| | **1st Quartile** | **Median** | **3rd Quartile** | **Range** |
|---|---|---|---|---|
| Days between HIV Diagnosis & Set-point Viral Load Test | 3 | 20 | 245 | -3,333 to 8,181 |

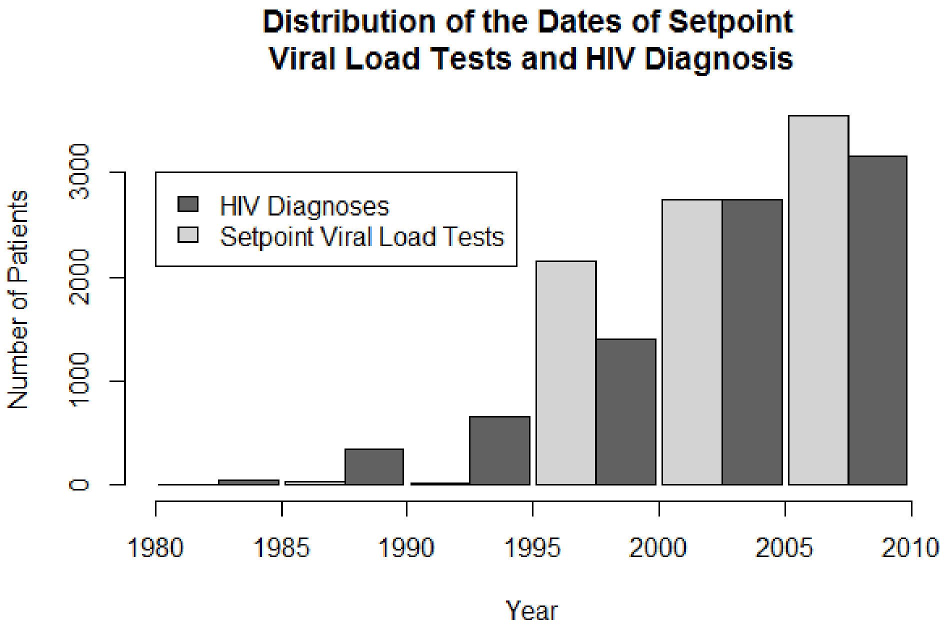


**Figure S4** – Histogram of the dates of HIV diagnosis (dark grey) and set-point viral load tests (light grey) in our subtype B dataset (*n=8,483*).

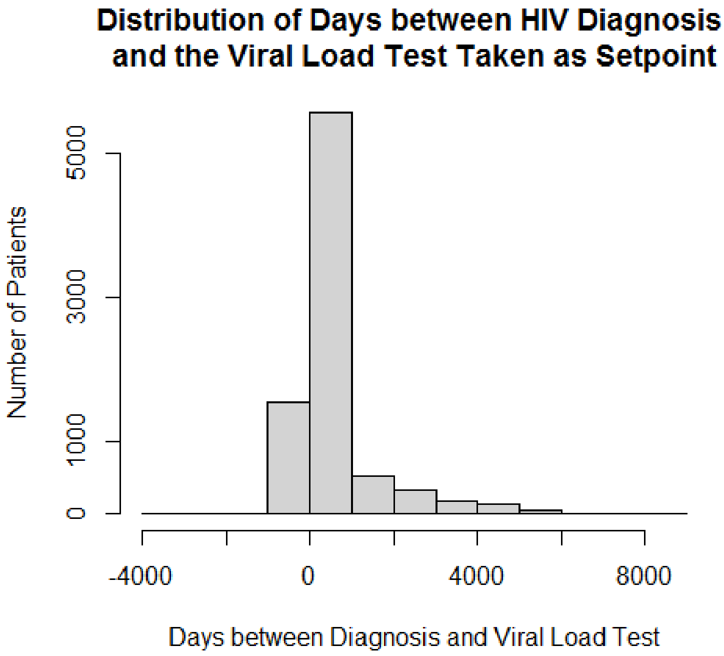


**Figure S5** – Histogram of the number of days between HIV diagnosis and set-point viral load test in our subtype B dataset (*n=8,483*).
